# Do Early Aortic Remodelling Patterns at 6 Months Predict Mid-Term Outcomes After Frozen Elephant Trunk for Chronic Aortic Dissection?

**DOI:** 10.1093/icvts/ivag046

**Published:** 2026-02-10

**Authors:** Sho Akita, Yoshiyuki Tokuda, Akinori Tamenishi, Yasumoto Matsumura, Akitaka Hayakawa, Masato Mutsuga

**Affiliations:** Department of Cardiac Surgery, Nagoya University Graduate School of Medicine, Nagoya, Aichi 466-8560, Japan; Department of Cardiovascular Surgery, Yokkaichi Municipal Hospital, Yokkaichi, Mie, 510-8567, Japan; Department of Cardiac Surgery, Nagoya University Graduate School of Medicine, Nagoya, Aichi 466-8560, Japan; Department of Cardiovascular Surgery, Yokkaichi Municipal Hospital, Yokkaichi, Mie, 510-8567, Japan; Department of Cardiovascular Surgery, Yokkaichi Municipal Hospital, Yokkaichi, Mie, 510-8567, Japan; Department of Cardiovascular Surgery, Yokkaichi Municipal Hospital, Yokkaichi, Mie, 510-8567, Japan; Department of Cardiac Surgery, Nagoya University Graduate School of Medicine, Nagoya, Aichi 466-8560, Japan

**Keywords:** frozen elephant trunk, chronic aortic dissection, aortic remodeling

## Abstract

**Objectives:**

To determine whether CT within 6 months after total arch replacement (TAR) with a frozen elephant trunk (FET) for chronic aortic dissection predicts mid-term outcomes and informs the timing of distal treatment.

**Methods:**

We analysed 56 consecutive patients who underwent TAR with FET at 2 centres (2009-2022) and had evaluable 6-month postoperative CT. Early remodelling was defined as the change from baseline to 6 months in the maximal outer-to-outer diameter of the proximal descending thoracic aorta at Level A (Ishimaru zone 3, 20 mm distal to the left subclavian artery, measured on centreline-orthogonal reconstructions). Patients were classified as early positive remodelling (EPR; no increase or a decrease) or early negative remodelling (ENR; ≥1-mm increase). Prespecified outcomes were distal aortic reintervention, distal stent graft-induced new entry (dSINE), and overall survival.

**Results:**

Mean follow-up was 5.4 years (standard deviation 3.7). Distal reintervention was required in 36/56 patients (64%). At 5 years, freedom from distal reintervention was higher with EPR than with ENR (44.6% vs 6.2%; *P* = .003). dSINE occurred in 26/56 patients (46.4%); 5-year dSINE-free survival was 65.1% (95% CI, 39.6-81.9) with EPR versus 18.2% (95% CI 5.9-35.2) with ENR (*P* = .008). Overall, 5-year survival for the cohort was 80.0% (95% CI 64.7-89.2). Among ENR patients, 5-year survival was 0% with conservative management versus 40.5% with distal intervention (*P* < .001); within EPR, 5-year survival was 65.9% with conservative management versus 85.7% with reintervention (*P* = .210).

**Conclusions:**

A 6-month CT provides simple, actionable risk stratification after TAR with FET for chronic aortic dissection. Absence of EPR identifies a high-risk subgroup (ENR) that warrants closer surveillance and timely distal intervention, optimizing follow-up intensity and treatment timing.

## Graphical abstract

**Figure:**
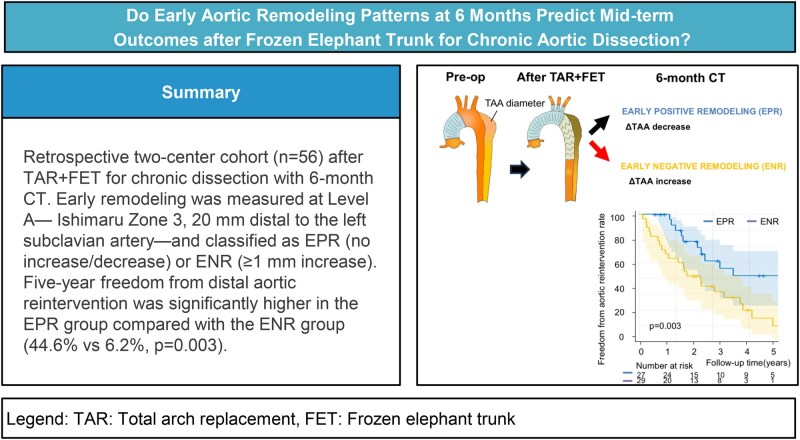

## INTRODUCTION

In patients with chronic aortic dissection, total arch replacement (TAR) with a frozen elephant trunk (FET) is an established surgical option, particularly for those with residual dissection after prior type A repair or anatomy unsuitable for thoracic endovascular aortic repair (TEVAR). This technique reconstructs the aortic arch and provides structural support to the proximal descending aorta, while simultaneously promoting favourable remodelling of the distal aorta.^1–5^ This hybrid approach addresses arch pathology and facilitates true-lumen expansion in the descending thoracic aorta, producing geometric changes that make it an attractive treatment option for extensive aortic dissections.

While the benefits of FET in acute type A aortic dissection are well-established, outcomes in chronic dissection remain less predictable. Approximately 30%-60% of patients with chronic dissection require reintervention following FET due to progressive aneurysmal degeneration or distal stent graft-induced new entry (dSINE) tears.^1^^,^^5^ This apparent paradox—whereby a procedure intended to induce favourable remodelling shows limited long-term efficacy in mature dissections—underscores our incomplete understanding of the biomechanical determinants of successful aortic remodelling.^3^^,^^6^ As a result, surveillance and intervention strategies remain largely empirical rather than guided by validated predictors of remodelling success. To address this gap, we analysed early postoperative CT to characterize remodelling after FET in chronic dissection, hypothesizing that morphological changes detectable at 6 months would predict mid-term outcomes; specifically, we sought to classify early remodelling phenotypes using quantitative imaging, evaluate their prognostic value for adverse aortic events, and define morphological thresholds to guide the timing of distal reintervention.

## METHODS

### Study design and patient selection

This retrospective cohort study evaluated aortic remodelling after FET procedures for chronic aortic dissection at 2 tertiary cardiac surgery centres. Among 260 patients who underwent TAR with FET between January 2009 and December 2022, 56 met the inclusion criteria of chronic dissection (≥3 months from initial onset; mean interval, 5.2 years [SD 3.9]) and availability of 6-month postoperative CT imaging. The primary surgical indication was aneurysmal dilatation of the proximal descending thoracic aorta (mean maximal diameter, 56.9 mm [SD 7.8]).

Exclusion criteria included acute dissection (<3 months), incomplete imaging follow-up at 6 months, and patients who died within the first 6 months postoperatively. The patient selection process is shown in **Figure S1**. All patients provided written informed consent for surgery and subsequent follow-up imaging.

### Surgical technique

All operations were performed under high moderate hypothermic circulatory arrest with pharyngeal temperature maintained between 24 and 28°C with bilateral antegrade selective cerebral perfusion.^7^ Arterial cannulation was via the right axillary artery in all cases.

The FET component comprised either hand-crafted Gianturco Z-stent constructs (*n* = 6; mean diameter 20.0 mm [SD 1.2], range 18-22 mm) or commercial Frozenix open stent grafts (Japan Lifeline, Tokyo, Japan; *n* = 50; mean diameter 25.8 mm [SD 3.6], range 18-35 mm). Mean insertion depth from the left subclavian artery origin was 10.8 cm [SD 2.6] (range 6-15 cm). In all cases, the FET was deployed in the true-lumen to seal the primary entry at the proximal descending thoracic aorta (Ishimaru zone 3), without fenestration or septectomy.

For stent graft diameter selection, we used a commonly adopted target of approximately 110% of the true-lumen diameter or 90% of the outer aortic diameter at the intended landing zone. In chronic dissection, because true-lumen and outer diameters often diverge, we adopted true-lumen perimeter-based sizing: the centreline-orthogonal perimeter was converted to an equivalent diameter (perimeter/*π*) and modest oversizing was applied. FET stent graft length was selected from the planned anastomosis site as the shortest centreline distance that achieved distal-end straightening (coaxial alignment) while ensuring that the proximal edge would not extend above the aortic valve level upon deployment. Concomitant procedures were undertaken as indicated, including coronary artery bypass grafting (*n* = 1), aortic valve replacement (*n* = 2), and aortic root replacement (*n* = 4).

### Follow-up protocol

Clinical follow-up was conducted through institutional outpatient clinics in collaboration with referring cardiologists and cardiac surgeons. Contrast-enhanced CT angiography was performed routinely before hospital discharge, at 6 months, at 12 months, and annually thereafter according to institutional protocols. The overall imaging follow-up compliance rate was 94.6% at 24 months. Clinical outcomes and imaging data were retrospectively reviewed from electronic medical records, with particular attention to the occurrence of dSINE and requirement for reintervention.

### Morphological analysis

All CT datasets were analysed with dedicated 3D vascular analysis software (3mensio Vascular, Photron M&E Solutions Inc., Tokyo, Japan). For each patient, a centreline was generated, and diameters were measured on true short-axis planes perpendicular to the centreline using outer-to-outer callipers to ensure reproducibility.

**Figure 1.**
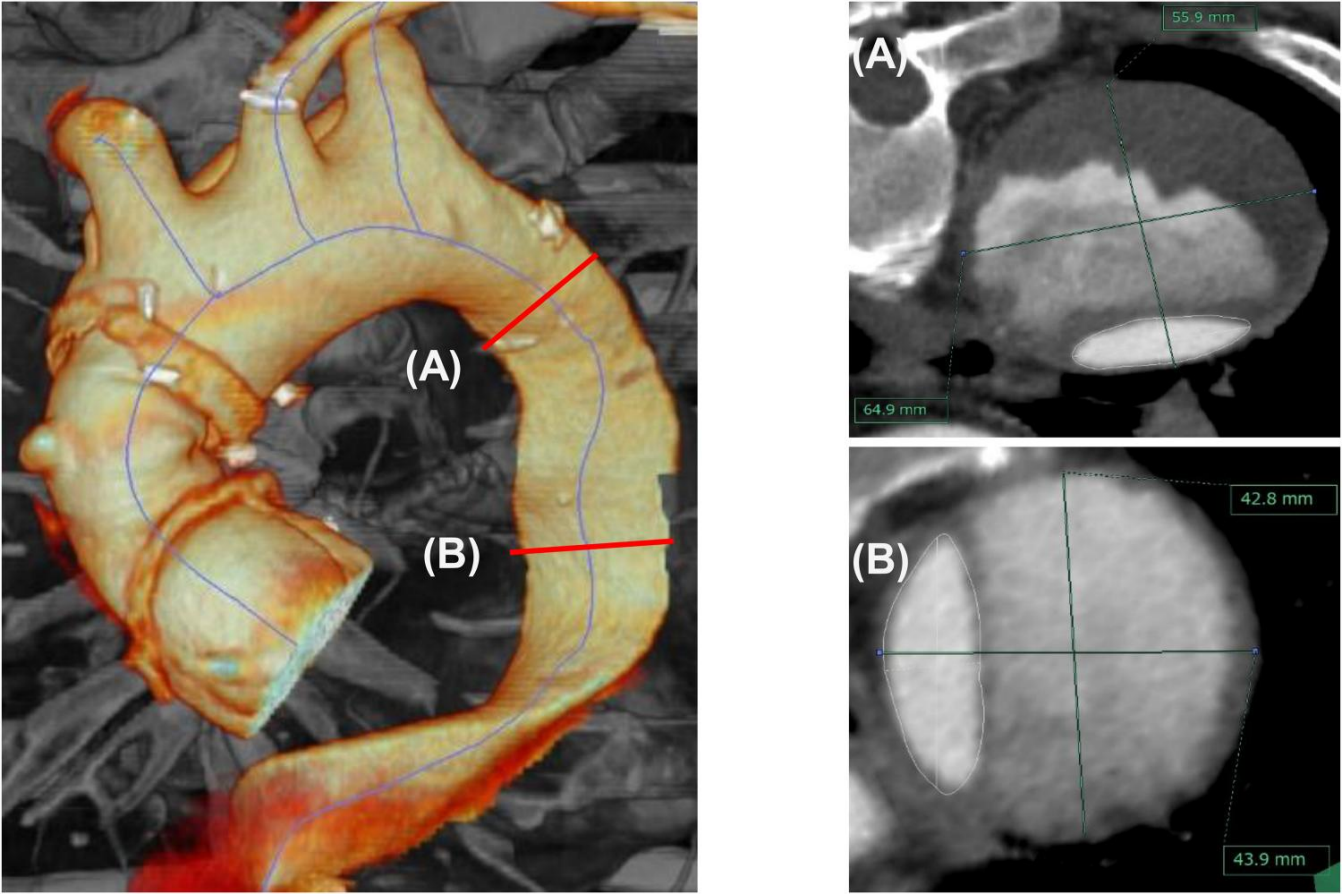
Standardized Aortic Measurement Levels. Left: 3D CT angiography showing level A in the proximal descending thoracic aorta (Ishimaru zone 3, 20 mm distal to the left subclavian artery) and level B in the mid-descending thoracic aorta (Ishimaru zone 4, at the level of the left lower pulmonary vein). Right: corresponding centreline-orthogonal cross-sections at level A (top) and level B (bottom) with outer-to-outer maximal aortic diameters.

In accordance with contemporary EACTS/STS aortic guidelines, aortic diameters were referenced to the Ishimaru zone classification. Two standardized levels were used in the present study^7^:

Level A was defined in the proximal descending thoracic aorta corresponding to Ishimaru zone 3, located 20 mm distal to the origin of the left subclavian artery along the aortic centreline (**Figure 1**). This level represents the target aneurysmal segment and served as the primary assessment level.

Level B was defined within Ishimaru zone 4 in the mid-descending thoracic aorta at the level of the left lower pulmonary vein, which was selected as a reproducible anatomical landmark within this relatively long segment and served as a reference level.

At each level, total aortic, true-lumen, and false-lumen diameters were recorded. Communication between the true and false lumens was assessed as previously described.^8^ For descriptive purposes, the proximal descending thoracic aorta was defined as Ishimaru zone 3, the mid-to-distal descending thoracic aorta as Ishimaru zone 4, and the abdominal aorta as the segment extending from the coeliac axis to the aortic bifurcation.

The FET angle was defined as the angle formed between the native aorta and the distal portion of the FET prosthesis.^9^ The level of the distal FET implantation site was specified according to the corresponding thoracic vertebral level (TH) where the stent graft ended. The FET oversizing ratio was calculated at the stent deployment site based on the true-lumen diameter derived from perimeter measurements on centreline true short-axis imaging.

### Definition of early remodelling patterns

Early aortic remodelling was defined as the change from baseline (preoperative) to 6-month postoperative CT at Level A (Ishimaru zone 3; 20 mm distal to the LSCA), measured on centreline true short-axis imaging. Six-month postoperative CT scans were ECG-gated in 55 of 56 patients (98.2%).

Patients were stratified at 6 months into 2 groups: early positive remodelling (EPR), defined as stable or decreased maximal diameter of the proximal descending thoracic aorta at Level A (Ishimaru zone 3), and early negative remodelling (ENR), defined as a ≥ 1 mm increase in diameter from baseline at the same level. All measurements were performed independently by 2 observers blinded to clinical outcomes, and interobserver agreement was quantified using intraclass correlation coefficients.

### Clinical outcomes

The primary outcome was reintervention-free survival at 5 years after FET, defined as the time from the index operation to the first distal aortic reintervention, including TEVAR, open thoraco-abdominal repair, or visceral debranching. Patients were censored at death or at the last clinical or imaging follow-up. Secondary outcomes included dSINE-free survival at 5 years, overall survival at 5 years, and longitudinal changes in aortic diameter at predefined measurement levels.

### Statistical analysis

Descriptive statistics were used to summarize the data. Continuous variables are reported as mean (standard deviation) for normally distributed data or median [interquartile range] for non-normally distributed data; categorical variables are presented as counts and percentages. Normality was assessed with the Shapiro-Wilk test. Between-group comparisons used the unpaired *t* test for normally distributed data or the Mann-Whitney *U* test for skewed data; categorical variables were compared with the chi-square test or Fisher’s exact test, as appropriate. *P*-values are reported to 3 decimal places, except when *P* < .001.

Longitudinal changes in aortic diameter from baseline (Δdiameter, mm) were evaluated at 2 prespecified levels—Ishimaru zone 3 (proximal descending thoracic aorta) and Ishimaru zone 4 (mid-to-distal descending thoracic aorta)—using linear mixed-effects models. For each level, fixed effects were time (months from baseline), remodelling group (EPR, early positive remodelling; ENR, early negative remodelling), and their interaction, with a patient-specific random intercept. The interaction term was the primary effect of interest. Estimates are reported with standard errors, *t* statistics, and *P-*values.

Time-to-event end-points (freedom from distal reintervention, freedom from dSINE, and overall survival) were analysed using Kaplan-Meier curves with log-rank tests. Complementary Cox models provided hazard ratios (HRs) with 95% CIs for remodelling group and treatment strategy. The proportional-hazards assumption was evaluated using Schoenfeld residuals. Results are presented as HR with 95% CI and 2-sided *P*-values; statistical significance was defined as *P* < .05. No multivariable (adjusted) analyses were performed.

All analyses were performed using EZR (Saitama Medical Center, Jichi Medical University, Saitama, Japan), a graphical user interface for R (The R Foundation for Statistical Computing, Vienna, Austria).^10^

This retrospective study was approved by the Institutional Review Board of Nagoya University Graduate School of Medicine (IRB 655-2) and the Institutional Review Board of Yokkaichi Municipal Hospital (IRB 2024-36), with the requirement for individual informed consent waived. The study was conducted in accordance with the principles of the Declaration of Helsinki and the WMA Declaration of Taipei regarding the use and storage of health-related data. Clinical and imaging data were obtained from existing institutional databases, anonymized prior to analysis, and used solely for the purposes of this study. No new biobank or collection of biological material was established for this research, and ongoing governance of institutional databases is overseen by the respective research ethics committees.

## RESULTS

### Patient characteristics

Patient characteristics are shown in **Table 1**. The study cohort comprised 56 patients with chronic aortic dissection who underwent FET procedures, with a mean age of 62.6 ± 8.4 years at the time of surgery. The majority of patients (44/56, 78%) had previously undergone hemiarch replacement for acute type A aortic dissection; the remainder had chronic type B aortic dissection that was anatomically unsuitable for TEVAR. The interval between the index dissection and the FET procedure was 5.2 years (SD 3.9; range 5-232 months). Communication between the true and false lumens was present in the proximal descending thoracic aorta (Ishimaru zone 3) in 56/56 patients (100%), in the mid-to-distal descending thoracic aorta (Ishimaru zone 4) in 4/56 (7.1%), and in the abdominal aorta in 51/56 (91.1%). Imaging follow-up was complete for all patients at 1 and 6 months postoperatively. Mean clinical follow-up was 5.4 years (SD 3.7; range 0.6-15.1 years).

**Table 1.** Patient characteristics.

Variables	ALL (*N* = 56)	EPR (*N* = 27)	ENR (*N* = 29)	*P*-value
Age (years)	62.6 ± 8.4	62.5 ± 8.3	62.8 ± 8.6	.89
Male	40 (71)	16 (59)	24 (83)	.053
Hypertension	44 (76)	24 (89)	20 (69)	.072
Diabetes mellitus	8 (14)	4 (15)	4 (14)	.91
Hyperlipidaemia	18 (32)	10 (37)	8 (28)	.45
BMI	24.2 ± 3.5	23.3 ± 3.5	24.6 ± 3.5	.27
Smoking	35 (63)	15 (56)	20 (69)	.31
Renal insufficiency	15 (27)	8 (30)	7 (24)	.65
Dialysis dependent	2 (4)	1 (4)	1 (3)	.96
Cerebral vascular disease	9 (16)	6 (22)	3 (10)	.23
Post type A dissection repair	44 (79)	20 (74)	24 (83)	.44
Marfan	5 (9)	3 (11)	2 (7)	.52
Years since primary dissection	5.2 ± 4.0	3.4 ± 3.1	6.9 ± 3.9	<.001
Aortic diameter at level A (mm)	56.8 ± 7.8	56.5 ± 8.6	57.1 ± 7.2	.74
Aortic diameter at level B (mm)	50.2 ± 8.3	46.1 ± 7.8	54.0 ± 7.0	<.001
FET size (mm)	25.3 ± 4.0	25.6 ± 4.2	25.1 ± 3.8	.68
FET length (cm)	11.0 ± 2.5	11.1 ± 2.8	10.8 ± 2.3	.61
Handmade FET	6 (18)	3 (11)	3 (10)	1
FET angle (°)	23.1 ± 13.3	22.4 ± 13.8	23.8 ± 13.1	.72
Landing level of FET (TH level)	6.9 ± 1.2	6.7 ± 1.4	7.1 ± 0.9	.36
FET oversizing (%)	6.2 ± 13.1	7.8 ± 14.6	4.9 ± 11.8	.41

### Early remodelling patterns and morphological changes

At 1 month postoperatively, complete thrombosis of the stented segment was observed in 38 patients (67.9%), while 18 patients (32.1%) demonstrated persistent false-lumen patency. Early evidence of favourable remodelling was already apparent in 16 patients (28.6%).

By 6 months, the cohort diverged into 2 phenotypes: EPR in 27/56 (48.2%) and ENR in 29/56 (51.8%). In univariable analysis, total aortic diameter at Level B was associated with ENR (HR 1.05; 95% CI 1.01-1.10; *P* = .018), and longer duration of chronic dissection was also significant (**Table 1**).

### Reintervention requirements

**Figure 2.**
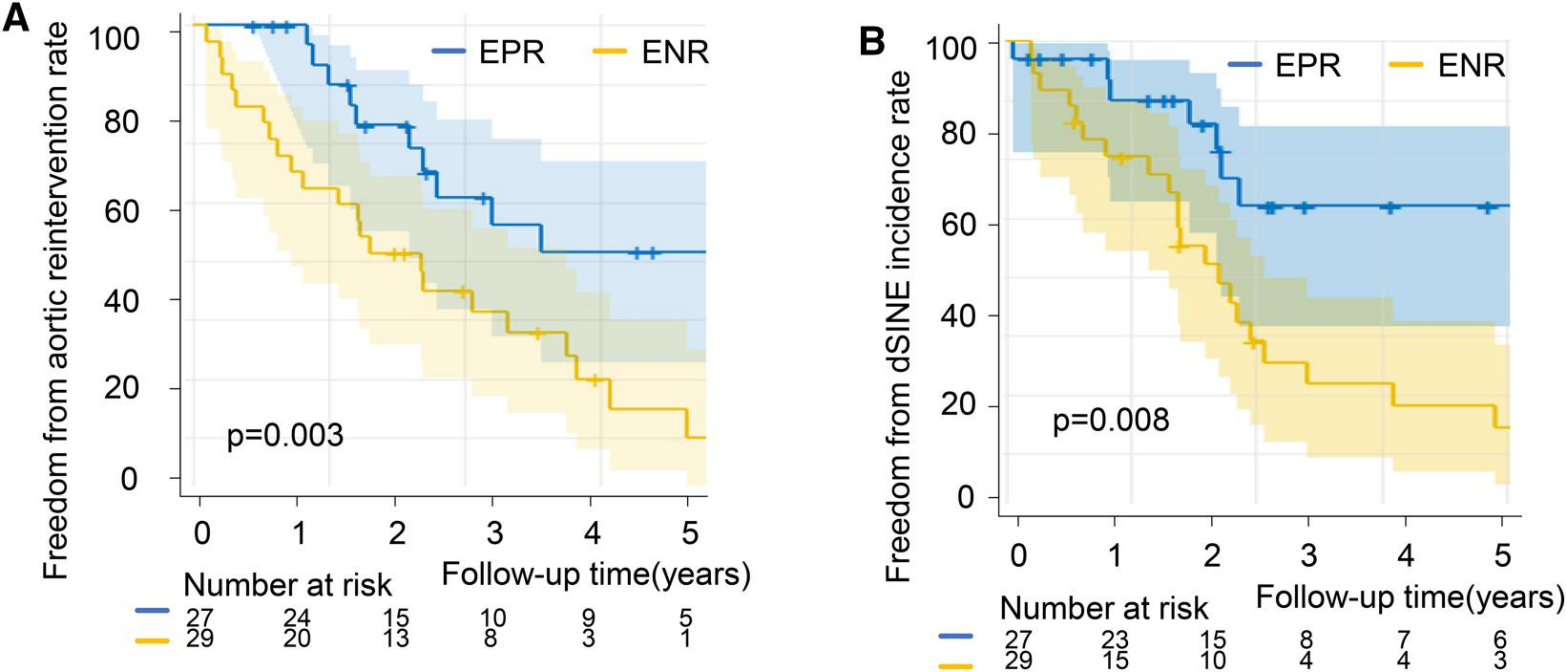
Freedom From Aortic Reintervention and dSINE by Early Remodelling Phenotype. Kaplan-Meier curves comparing EPR (blue) and ENR (yellow). (A) Freedom from aortic reintervention: ENR was significantly lower than EPR (B) freedom from dSINE: ENR was significantly lower than EPR. Abbreviations: dSINE, distal stent-graft-induced new entry; ENR, early negative remodelling; EPR, early positive remodelling.

Thirty-six of 56 patients (64%) required distal aortic reintervention at a mean interval of 2.1 years (SD 1.5) after FET implantation. Early reintervention within the first postoperative year occurred in 9 patients (7 open surgical repairs, 2 TEVAR). A further 20 patients underwent late reintervention (6 open surgical repairs, 14 TEVAR). Five-year reintervention-free survival for the entire cohort was 24.3% (95% CI 11.8-39.1). At 5 years, reintervention-free survival was 6.2% (95% CI 4.6-24.0) in the ENR group and 44.6% (95% CI 21.4-65.5) in the EPR group (*P* = .003) (**Figure 2**).

### Distal stent-graft-induced new entry

dSINE occurred in 26/56 (46.4%) patients at a mean of 27 ± 18 months after FET (range 3-67 months). Freedom from dSINE was 42.5% at 3 years and 38.3% at 5 years in the overall cohort. At 5 years, dSINE-free survival was 65.1% (95% CI 39.6-81.9) in the EPR group and 18.2 (95% CI 5.9-35.2) in the ENR group (*P* = .008) (**Figure 2**).

**Figure 3.**
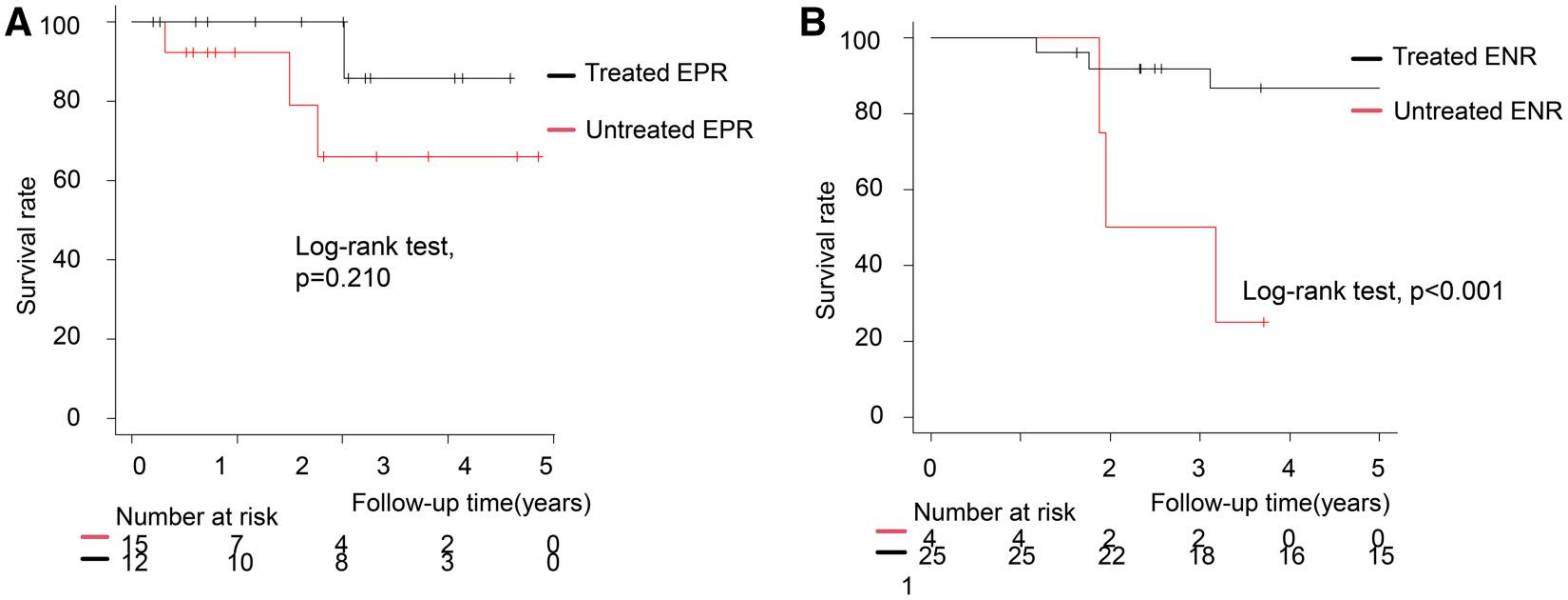
Overall Survival by Early Remodelling Phenotype and Treatment Status. (A) EPR subgroup: treated (black) vs untreated (red); survival curves were not significantly different (log-rank *P* = .210). (B) ENR subgroup: treated (black) vs untreated (red); untreated ENR showed markedly worse survival (log-rank *P* < .001). Abbreviations: ENR, early negative remodelling; EPR, early positive remodelling.

There were 12 deaths overall, 8 (66.7%) attributable to aortic-related causes. Freedom from all-cause mortality was 90.3% (95% CI 78.2-95.8) at 3 years and 80.0% (95% CI 64.7-89.2) at 5 years. Five-year overall survival did not differ significantly between EPR and ENR (83.6% vs 77.2%; *P* = .520). In subgroup analyses, within EPR 5-year survival was 85.7% in patients who underwent reintervention and 65.9% in those managed conservatively (*P* = .210). Within ENR, patients without reintervention had 0% survival at 5 years, whereas those who underwent distal treatment had 40.5% survival (*P* < .001) (**Figure 3**).

Aorto-oesophageal fistula (AEF) developed in 3 patients (10.3% of the ENR group), all requiring emergency surgical intervention. No cases of AEF were observed in patients with EPR.

### Longitudinal morphological assessment

#### Level a diameter trajectory

Preoperatively, the mean aortic diameter at Level A was 56.8 mm (SD 7.8), increased transiently to 58.2 mm (SD 7.6) immediately postoperatively, then decreased to 54.9 mm (SD 10.3) at 6 months and 50.5 mm (SD 10.6) at 1 year, thereafter stabilizing around 51 mm. A statistically significant reduction from baseline was observed from 1 year onward (*P* = .024).

#### Group-wise change (linear mixed-effects model)

**Figure 4.**
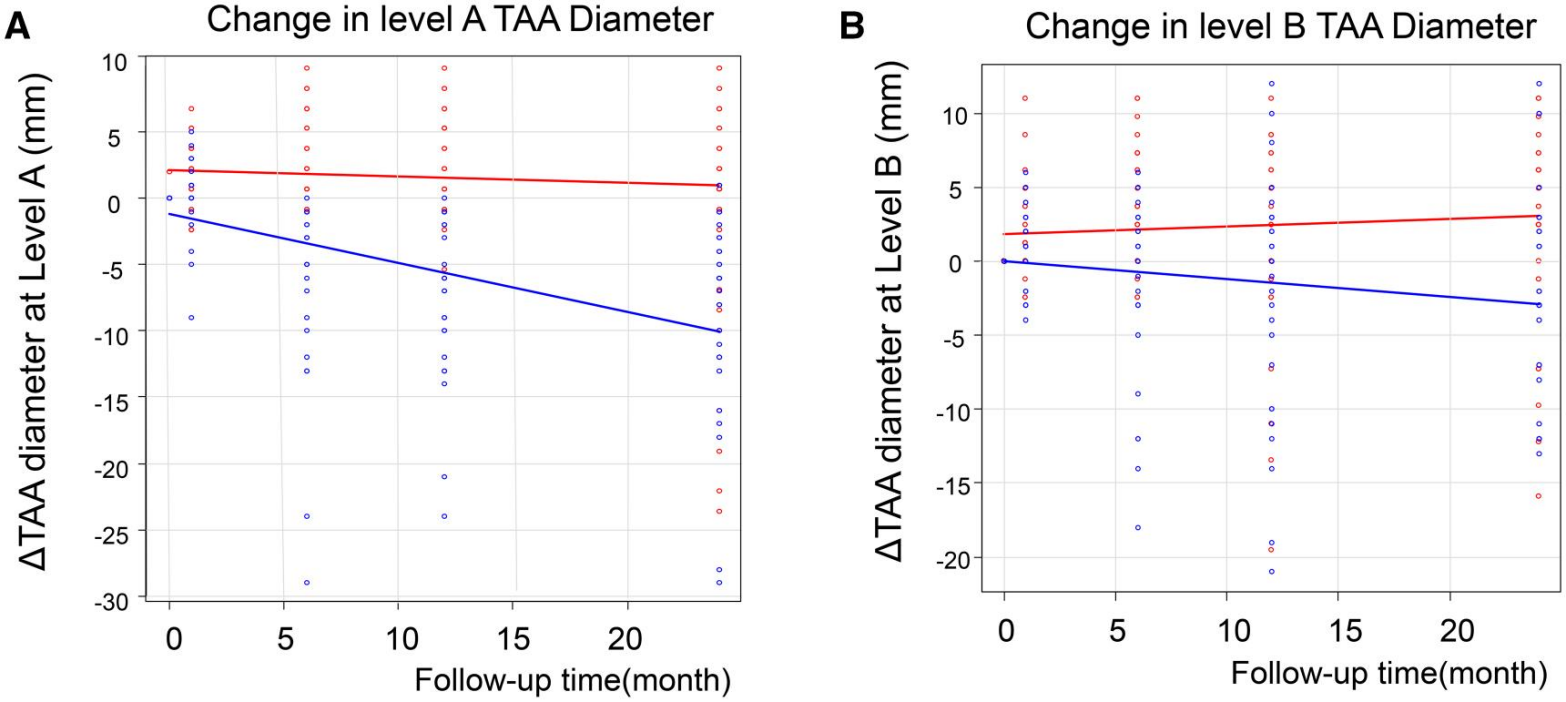
Change From Baseline in TAA Diameter (ΔTAA, mm) After FET by Remodelling Phenotype. (A) Level A (proximal descending thoracic aorta) and (B) level B (mid-descending thoracic aorta) at 0, 1, 6, 12, and 24 months. EPR (blue) shows progressive regression, whereas ENR (red) shows little reduction or expansion. Abbreviations: ENR, early negative remodelling; EPR, early positive remodelling.

The mixed-effects analysis showed distinct trajectories by remodelling group: EPR exhibited a consistent diameter decrease averaging −0.37 mm/month, whereas ENR showed minimal change (−0.03 mm/month). The 6-month change in diameter differed between the groups (EPR 2.0 mm [SD 3.5] vs ENR −2.2 mm [SD 6.9]; *P* = .013) (**Figure 4**).

## DISCUSSION

This study demonstrates that aortic remodelling patterns assessed by CT at 6 months after FET implantation are strong predictors of mid-term outcomes in patients with chronic aortic dissection. Stratification into the EPR and ENR groups, based on changes in proximal descending aortic diameter, revealed distinct morphological trajectories closely associated with long-term clinical events. These findings are consistent with emerging evidence that morphological response patterns following hybrid aortic interventions in chronic dissection can serve as reliable surrogates for long-term clinical success.^11^^,^^12^

Two features of the EPR group—shorter dissection duration and smaller mid-descending aortic diameter—suggest a less advanced pathological substrate that favours true-lumen expansion after FET, supporting earlier referral for arch-FET procedures. Prospective studies are needed to define optimal timing and diameter thresholds for intervention. Our previous work suggested approximately 45 mm as a candidate cut-off for dSINE risk.^13^

Patients in the EPR group demonstrated significant regression of the target thoracic aortic aneurysm, reflecting effective true-lumen expansion and false-lumen thrombosis. This favourable morphological response aligns with the therapeutic goal of FET in chronic dissection, where restoration of physiological flow patterns and elimination of competitive false-lumen flow are particularly challenging.^1^^,^^5^^,^^6^^,^^14^ In contrast, ENR patients—comprising over half of the cohort—showed minimal or no diameter reduction despite technically successful procedures. This lack of early remodelling was associated with markedly higher rates of dSINE, more frequent reinterventions, and the occurrence of AEF exclusively within the ENR group. The high incidence of dSINE in our series (46.4%) is consistent with reported rates in chronic dissection cohorts undergoing FET, which range from 30% to 60% depending on follow-up duration and patient selection criteria.^9^^,^^15–18^ Notably, whereas no untreated ENR patient survived to 5 years, survival among treated ENR and EPR patients was substantially better. Even within the EPR group, conservatively managed patients showed a non-significant trend towards lower 5-year survival compared with those who underwent reintervention (65.9% vs 85.7%; *P* = .21). These findings underscore the need for risk stratification and timely distal intervention in this challenging population and suggest that FET alone is rarely definitive therapy for chronic dissection.

These findings underscore the distinct biomechanical milieu of chronic dissection, fundamentally different from other aortic diseases.^1^^,^^6^^,^^19^ Progressive thickening and fibrotic transformation of the intimal flap, together with a rigid false-lumen wall, resist stent graft radial expansion.^20^ Biomechanical studies show time-dependent stiffening and reduced compliance versus acute dissections, altering responses to endovascular therapy.^21^^,^^22^ Consequently, false-lumen perfusion persists, circumferential wall stress remains elevated, and aneurysmal degeneration progresses despite stent deployment. Over months to years, fibrosis, calcification, and mechanical stiffening further entrench this substrate, limiting the flow-redirecting mechanisms required for durable FET outcomes.^23^^,^^24^ These chronic structural changes create an inherently unfavourable substrate for the flow-redirecting mechanisms that underpin successful FET outcomes.

The high dSINE incidence in the ENR group merits emphasis, and our overall rate (46%) exceeded prior reports—likely reflecting longer chronicity and larger mid-descending diameters in this cohort.^13^^,^^15^^,^^16^ The concentration of dSINE among patients without early remodelling suggests that the same factors impeding false-lumen thrombosis—wall rigidity and persistent pressurization—also predispose to device-related complications. Early morphological response thus appears to index underlying aortic compliance, offering prognostic value beyond static anatomical measurements.

The development of AEF exclusively in the ENR group is particularly concerning, as this complication carries an extremely high mortality rate despite urgent intervention.^18^^,^^25^ In the setting of chronic dissection, AEF likely reflects multifactorial mechanisms, including persistent false-lumen pressurization, chronic inflammatory remodelling, and reduced compliance of a fibrotic aortic wall that may predispose to erosive injury.^26^^,^^27^ The restriction of AEF to patients without early remodelling supports the hypothesis that sustained false-lumen pressure and localized wall-stress concentration contribute to its development in this population.

From a clinical management perspective, incorporating 6-month postoperative CT into routine follow-up identifies high-risk ENR patients and opens a window to optimize intervention strategies.^28^^,^^29^ This shifts care from reactive to predictive management in chronic aortic dissection. For ENR, we recommend intensified surveillance (imaging every 3-6 months rather than annually) and consideration of earlier prophylactic distal intervention before aneurysmal progression.^29^ Such proactive management aims to prevent catastrophic complications and improves the safety of long-term care.

### Limitations

This 2-centre retrospective study (*n* = 56) is underpowered and prone to residual confounding. Attrition from 260 TAR + FET procedures to 62 chronic dissection cases and to 56 with evaluable 6-month CT may have introduced selection and survivor bias. Imaging protocols evolved over time (6-month CT ECG-gated in 55/56), and measurement error is possible despite standardization. Finally, no multivariable adjustment was performed.

## CONCLUSIONS

Early aortic remodelling patterns at 6 months after FET are strong predictors of outcomes in chronic aortic dissection. Patients with early negative remodelling had higher rates of dSINE, reintervention, and severe complications, with no survivors at 5 years when left untreated. Routine imaging at 6 months can identify high-risk patients and guide timely intervention, underscoring the need for early recognition and proactive management to improve long-term prognosis.

## Supplementary Material

ivag046_Supplementary_Data

## Data Availability

The data that support the findings of this study are available from the corresponding author, [S.A.], upon reasonable request.
