# Supplementary material for: Do Early Aortic Remodelling Patterns at 6 Months Predict Mid-Term Outcomes After Frozen Elephant Trunk for Chronic Aortic Dissection?
Source: Interdiscip Cardiovasc Thorac Surg. 2026 Feb 10;41(2):ivag046. doi: 10.1093/icvts/ivag046 (PMC12944824; doi:10.1093/icvts/ivag046)
Supplement: ivag046_Supplementary_Data [file ivag046_supplementary_data.pdf]

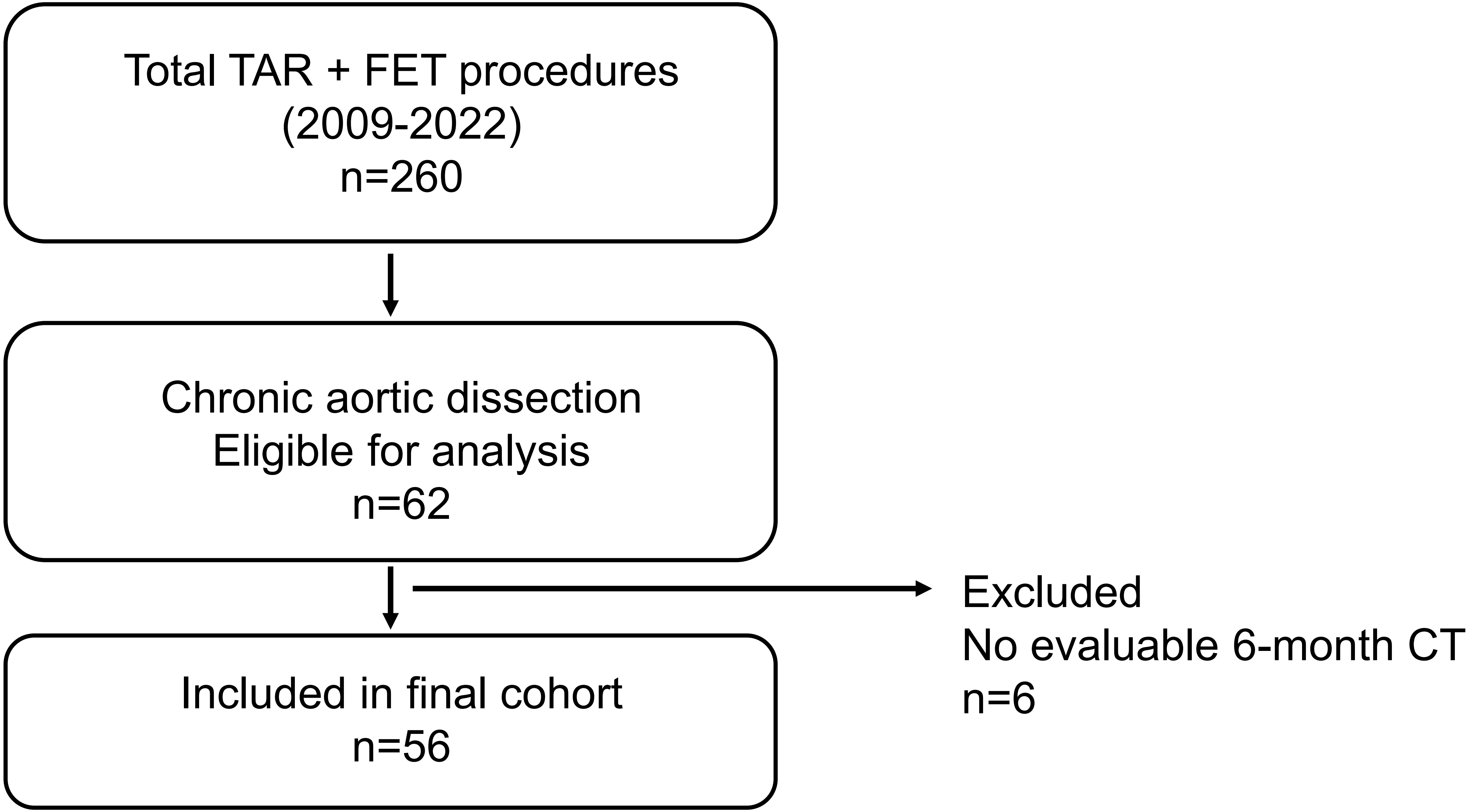
Total TAR + FET procedures
(2009-2022)
n=260
Chronic aortic dissection
Eligible for analysis
n=62
Excluded
No evaluable 6-month CT
n=6
Included in final cohort
n=56
